# Synergistic interaction of amphotericin B and betulinic acid against clinically important fungi: evidence from *in vitro* and *in silico* techniques

**DOI:** 10.1128/spectrum.03333-24

**Published:** 2025-05-16

**Authors:** Bence Rafael, Mónika Homa, Csilla Szebenyi, Csaba Vágvölgyi, Chetna Tyagi, Tamás Papp

**Affiliations:** 1 Department of Biotechnology and Microbiology, Faculty of Science and Informatics, University of Szeged 37442, Szeged, Hungary; 2 HUN-REN-SZTE Fungal Pathomechanisms Research Group, University of Szeged 37442, Szeged, Hungary; Agricultural Research Organization Volcani Center, Rishon LeZion, Israel

**Keywords:** Eagle effect, candidiasis, aspergillosis, mucormycosis, *in silico *molecular docking, mixed pore formation

## Abstract

Betulinic acid (BA), in combined application with amphotericin B, shows a synergistic effect against *Candida*, *Aspergillus*, *Scedosporium*, *Fusarium*, and *Mucorales* fungi at a concentration as low as 0.125 µg/mL. Amphotericin B showed slightly higher affinity towards BA than toward ergosterol, according to our *in silico* molecular docking results, explaining the observed Eagle effect. Moreover, it can bind both molecules simultaneously, suggesting the possibility of the formation of mixed pores, thus increasing the membrane-disrupting activity.

**IMPORTANCE:**

The rising incidence of invasive fungal infections, coupled with the emergence of antifungal resistance, presents a significant challenge in clinical settings. The inherent resistance of certain fungi to conventional antifungal agents, alongside the limitations posed by side effects and drug interactions, necessitates the exploration of alternative therapeutic strategies. This study highlights the potential of combining amphotericin B (AmB) with betulinic acid (BA) to enhance antifungal efficacy against clinically relevant pathogens, including *Candida albicans* and *Aspergillus fumigatus*, as well as mucormycosis-causing fungi. The results demonstrate the synergistic interactions between AmB and BA, which effectively inhibited fungal growth at lower concentrations and are within reported serum levels. *In silico* molecular docking studies further support the hypothesis that BA may facilitate AmB’s mechanism of action, potentially leading to increased pore formation in fungal membranes.

## OBSERVATION

The number of patients affected by invasive fungal infections is increasing. However, treatment is often complicated by the spread of strains resistant to antifungal agents. Additionally, several fungi, such as those causing mucormycosis (1), are inherently resistant to most antifungal agents or require higher doses for growth inhibition (2). Treatment options are further limited by incompatibility with antifungal medication, side effects, and underlying diseases.

Amphotericin B (AmB) is widely used due to its broad efficacy. Its lipid formulations improve solubility and reduce nephrotoxic side effects, though not entirely (3). Betulinic acid (BA), a lupane-structured pentacyclic triterpene derived from betulin (4), is primarily known for its antitumor activity (5) but also exhibits antifungal properties (6). Its high lipophilicity allows easy diffusion through cell membranes (7) and crossing of the blood-brain barrier (4), making it potentially useful for treating cerebral mycosis. BA is still under preclinical studies, which suggest its potential use against different types of cancer (8).

In this study, we present the synergistic effect of AmB and BA against ten human pathogenic fungi. As rhino-orbito-cerebral form is one of the most frequent manifestations of mucormycosis and AmB is recommended as the only first-line agent to treat mucormycosis, we selected one strain of three mucormycosis-causing species, i.e., *Rhizopus oryzae* (CBS 109.939), *Rhizopus microsporus* (CBS 102.277), and *Lichtheimia corymbifera* (FSU 9682), and one strain of *Mucor lusitanicus* (CBS 277.49), which is a frequently used model organism to study fungal pathogenicity (1). One isolate of the most common mycosis-causing fungi, *Candida albicans* (CBS 562) (9) and *Aspergillus fumigatus* (NRRL 5109) (10), was also included. Since *Scedosporium* and *Fusarium* species are inherently less susceptible to AmB (11), two strains for these genera were selected with lower (i.e., *S. boydii* CBS 117410 and *F. solani* SZMC 11524) or higher (i.e., *S. aurantiacum* CBS 136046 and *F. solani* SZMC 11528) minimum inhibitory concentrations (MICs) of AmB. In the susceptibility tests, *Candida krusei* ATCC 6258 was used as a reference strain. Eagle effect was defined according to Valero et al. (12).

Antifungal susceptibility tests were conducted in 96-well microtiter plates following CLSI recommendations (13) in three biological replicates. Stock solutions of AmB and BA (both purchased from Merck, Darmstadt, Germany) were prepared in dimethyl sulfoxide and diluted with RPMI-1640 medium (Apollo Scientific, United Kingdom). Inocula (10⁴ spores or cells) were prepared in liquid RPMI-1640, and plates were incubated for 48 hours at 25°C for *M. lusitanicus* and 37°C for other fungi. To assess the sole effectiveness, AmB concentrations ranged from 0.125 to 256 µg/mL, while BA concentrations ranged from 0.125 µg/mL to 2 mg/mL. For combined testing, AmB and BA concentrations ranged from 0.125 to 64 µg/mL and 0.125 to 256 µg/mL, respectively. Fractional inhibitory concentration index (FICI) determined interaction types: synergistic (FICI ≤0.5), additive (0.5 < FICI ≤ 1), indifferent (1 < FICI ≤ 4), and antagonistic (FICI >4). When BA alone did not inhibit growth, twice the highest tested concentration was used for the FICI calculation (14).

**Fig 1:**
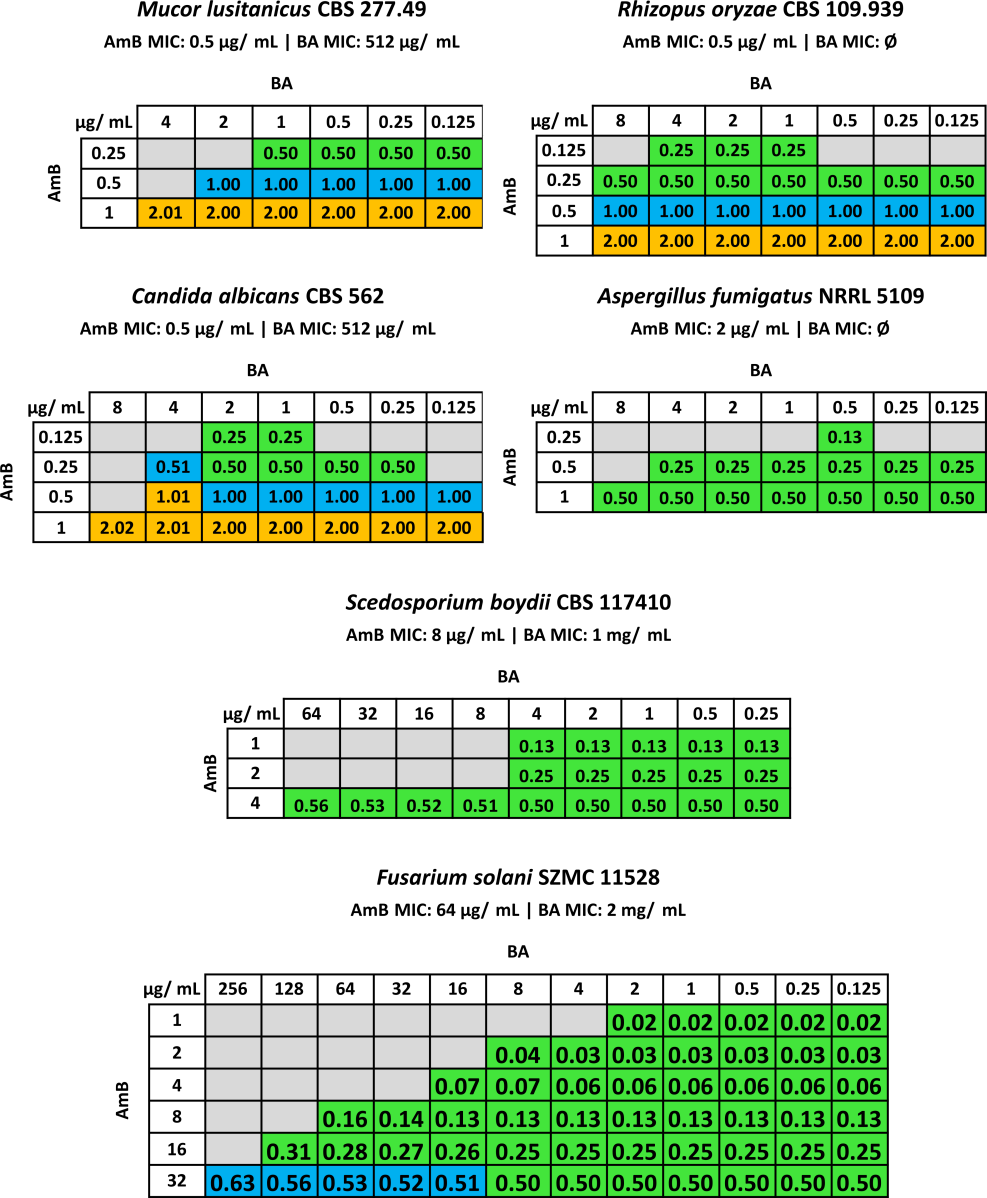
Minimal inhibitory concentrations (MICs) and combined synergistic application of AmB and BA in case of six pathogenic fungal strains indicated with the FICI values and its effect in the cells. For the FICI values, we interpreted FICI ≤0.5 as synergistic (green), 0.5 < FICI ≤ 1 as additive (blue), 1 < FICI ≤ 4 as indifferent (orange), and FICI >4 as antagonistic interactions. Gray color indicates those concentration combinations where the growth of the fungi was not inhibited.

MICs of AmB ranged from 0.25 to 64 µg/mL (Fig. 1, Fig. S1). Previously, BA MICs were reported for *A. fumigatus* at 16 µg/mL (15) and for *C. albicans* between 8 µg/mL (16) and 256 µg/mL (17). In our experiments, BAMICs were 512 µg/mL for *M. lusitanicus* and *C. albicans*, 1 mg/mL for *S. aurantiacum*, and 2 mg/mL for *S. boydii* and *F. solani* strains, but it proved to be ineffective against the other strains (Fig. 1, Fig. S1). Although BA is still in the preclinical testing phase, available serum concentrations have been reported in mice and rats, which were 4.2 and 0.68 µg/mL, respectively (9).

In combination with AmB, lower BA concentrations (0.125 to 4 µg/mL) were sufficient to inhibit all strains, falling within the reported serum concentrations (9). These AmB-BA combinations showed a synergistic effect across all strains (Fig. 1, Fig. S1). For *F. solani* SZMC 11528, growth inhibition was achieved with 16-fold less AmB and 16,000-fold less BA, and for *S. aurantiacum* CBS 136046, with 16-fold less AmB and 500-fold less BA in combination than their MICs alone. Notably, at higher BA concentrations, antagonism—and thus, the Eagle effect (12)—was observed for all strains.

**Fig 2:**
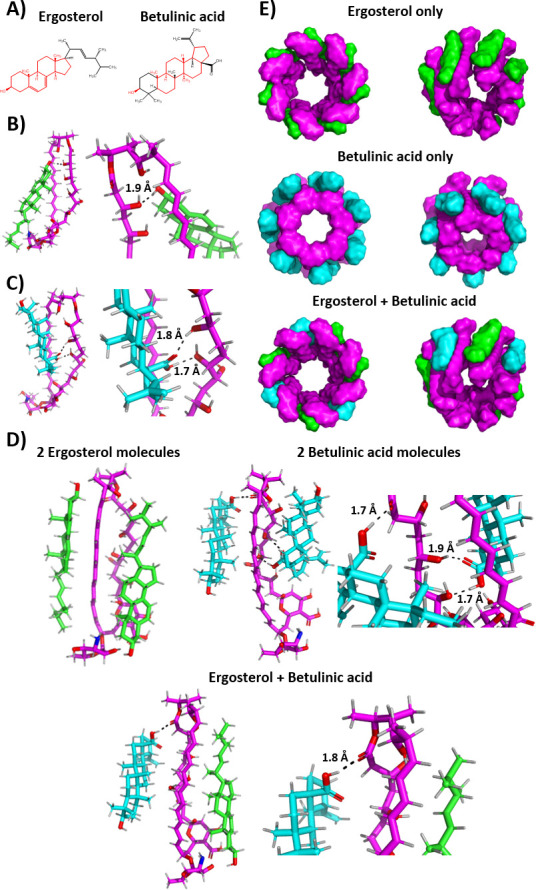
*In silico* molecular docking of amphotericin B to individual, multiple, and simultaneously to ergosterol and betulinic acid. (**A**) Structure of ergosterol and betulinic acid. Similarities in structure are highlighted with red. (**B**) Amphotericin B docking to ergosterol. 3D representation of the lowest energy interaction made by using PyMOL (22). Magenta: amphotericin B; green: ergosterol; black-dotted line: hydrogen bond. (**C**) Amphotericin B docking to betulinic acid. 3D representation of the lowest energy interaction made by using PyMOL (22). Magenta: amphotericin B; cyan: betulinic acid; black-dotted line: hydrogen bond. (**D**) Amphotericin B docking to multiple and simultaneously to ergosterol and betulinic acid. 3D representation of lowest energy interactions made by using PyMOL (22). Magenta: amphotericin B; green: ergosterol; cyan: betulinic acid; black-dotted line: hydrogen bond. (**E**) Prediction of the heptameric amphotericin B pores while binding only ergosterol, only betulinic acid, and both molecules simultaneously based on the work of Umegawa et al. (23) made by using M-ZDock (24). Magenta: amphotericin B; green: ergosterol; cyan: betulinic acid.

The antifungal effect of AmB is based on its binding to ergosterol (ERG), the main sterol component of the fungal membrane, which leads to the disruption of the cell membrane (18). BA and ERG have a similar core structure (Fig. 2A). Therefore, it is possible that BA can also act as a target for AmB, which can lead to the nonlinear efficacy of the compounds in combined application and could explain the observed Eagle effect. To address this matter, we conducted *in silico* molecular docking simulations of AmB to ERG and to BA (Table 1). Structural data files (SDF) of AmB (CID 5280965), BA (CID 64971), and ERG (CID 444679) were obtained from the PubChem database (https://pubchem.ncbi.nlm.nih.gov/). ORCA-readable files were generated using Avogadro 1.2.0 (19), followed by structure geometry optimization with ORCA 6.0.1. Docking of the geometry-optimized molecules was carried out with DOCKER (20, 21). For the individual docking experiments, we used the top five geometry-optimized structures, while for the multiple docking experiments, we used the best geometry-optimized structures of each molecule. Visualization and evaluation of *in silico* molecular docking was done using Avogadro and PyMOL (22).

**TABLE 1:** Predicted interaction energies and interactions of *in silico* molecular docking simulations of amphotericin B to ergosterol (ERG) and betulinic acid (BA) using ORCA DOCKER (21)

Ligands for binding with AmB	Interaction energy	Predicted interactions
Single ERG	−27.011290 kcal/mol	One hydrogen bond
Single BA	−27.930907 kcal/mol	Two hydrogen bonds
Two ERG	−17.700973 kcal/mol	Van der Waals interaction
Two BA	−15.232920 kcal/mol	Three hydrogen bonds
Single ERG +single BA	−18.465394 kcal/mol	One hydrogen bond (BA) + Van der Waals interaction (ERG)

Our simulations correctly predicted the experimentally known interaction between AmB and ERG (18) (Fig. 2B). Binding affinity between AmB and BA was slightly higher than between AmB and ERG (Table 1, Fig. 2B and C). We hypothesized that, due to the increased quantity of lipophilic compounds available for binding and BA’s diffusability through the membrane (8), it is possible that BA could act as a target for binding and help AmB’s pore formation ability either by the formation of pores consisting of BA and AmB only or by the formation of mixed pores consisting of BA, ERG, and AmB. This might be possible since each pore-forming AmB molecule requires two sterol molecules for binding (23). Hence, the number of pores can be increased and explain the synergistic effect.

Therefore, docking simulations of AmB were carried out in the presence of two ERG-, two BA-, and both molecules as well, where the simultaneous binding of both molecules had the lowest interaction energy (Table 1, Fig. 2D). These similar results suggest the possibility of AmB being able to bind not just multiple ERG molecules simultaneously (23), but also multiple BA molecules or even both types of molecules. Thus, all three types of pores (i.e., ERG+AmB, BA+AmB, and ERG+BA+AmB) (Fig. 2E) may be formed, resulting in significantly increased membrane-disrupting activity and explaining the synergistic effect.

At lower concentrations of BA, it may promote AmB aggregation below its critical oligomerization concentration by stabilizing intermolecular interactions and shifting the equilibrium toward oligomeric (4–8 molecules) and poly-aggregated states, which are more bioactive than monomeric AmB (23, 25). However, at higher concentrations of BA, it is possible that all binding sites on AmB are “masked” by BA, resulting in the inability of AmB to bind to the fungal membrane and potentially disrupting its oligomerization, rendering it inactive and leading to the observed Eagle effect. Further experiments are needed to ascertain its mechanism of action.

## SUPPLEMENTAL MATERIAL

Figure S1 spectrum.03333-24-s0001.pdf Results of all antifungal susceptibility tests performed in the study.

ASM does not own the copyrights to Supplemental Material that may be linked to, or accessed through, an article. The authors have granted ASM a non-exclusive, world-wide license to publish the Supplemental Material files. Please contact the corresponding author directly for reuse.
