# Supplementary material for: Synergistic interaction of amphotericin B and betulinic acid against clinically important fungi: evidence from in vitro and in silico techniques
Source: Microbiol Spectr. 2025 May 16;13(7):e03333-24. doi: 10.1128/spectrum.03333-24 (PMC12211039; doi:10.1128/spectrum.03333-24)
Supplement: Figure S1 — Results of all antifungal susceptibility tests performed in the study. [file spectrum.03333-24-s0001.pdf]

Fig S1

Mucor lusitanicus **CBS 277.49**

BA (columns) / AmB (rows)

µg/ mL	256	128	64	32	16	8	4	2	1	0.5	0.25	0.125
0.125												
0.25									0.50	0.50	0.50	0.50
0.5								1.00	1.00	1.00	1.00	1.00
1							2.01	2.00	2.00	2.00	2.00	2.00

AmB MIC: 0.5 µg/mL
BA MIC: 512 µg/mL

Rhizopus microsporus **CBS 102.277**

BA (columns) / AmB (rows)

µg/ mL	256	128	64	32	16	8	4	2	1	0.5	0.25	0.125
0.125						0.50	0.50	0.50	0.50	0.50	0.50	0.50
0.25					1.00	1.00	1.00	1.00	1.00	1.00	1.00	1.00
0.5					2.00	2.00	2.00	2.00	2.00	2.00	2.00	2.00
1						4.00	4.00	4.00	4.00	4.00	4.00	4.00

AmB MIC: 0.25 µg/mL
BA MIC: Ø

Rhizopus oryzae **CBS 109.939**

BA (columns) / AmB (rows)

µg/ mL	256	128	64	32	16	8	4	2	1	0.5	0.25	0.125
0.125							0.25	0.25	0.25			
0.25						0.50	0.50	0.50	0.50	0.50	0.50	0.50
0.5						1.00	1.00	1.00	1.00	1.00	1.00	1.00
1						2.00	2.00	2.00	2.00	2.00	2.00	2.00

AmB MIC: 0.5 µg/mL
BA MIC: Ø

Lichtheimia corymbifera **FSU 9682**

BA (columns) / AmB (rows)

µg/ mL	256	128	64	32	16	8	4	2	1	0.5	0.25	0.125
0.125									0.25	0.25	0.25	0.25
0.25						0.50	0.50	0.50	0.50	0.50	0.50	0.50
0.5						1.00	1.00	1.00	1.00	1.00	1.00	1.00
1						2.00	2.00	2.00	2.00	2.00	2.00	2.00

AmB MIC: 0.5 µg/mL
BA MIC: Ø

Aspergillus fumigatus **NRRL 5109**

BA (columns) / AmB (rows)

µg/ mL	256	128	64	32	16	8	4	2	1	0.5	0.25	0.125
0.125												
0.25										0.13		
0.5							0.25	0.25	0.25	0.25	0.25	0.25
1						0.50	0.50	0.50	0.50	0.50	0.50	0.50

AmB MIC: 2 µg/mL
BA MIC: Ø

Candida albicans **CBS 562**

BA (columns) / AmB (rows)

µg/ mL	256	128	64	32	16	8	4	2	1	0.5	0.25	0.125
0.125								0.25	0.25			
0.25							0.51	0.50	0.50	0.50	0.50	
0.5							1.01	1.00	1.00	1.00	1.00	1.00
1						2.02	2.01	2.00	2.00	2.00	2.00	2.00

AmB MIC: 0.5 µg/mL
BA MIC: 512 µg/mL

Scedosporium boydii **CBS 117410**

BA (columns) / AmB (rows)

µg/ mL	256	128	64	32	16	8	4	2	1	0.5	0.25	0.125
0.125												
0.25												
0.5												
1							0.13	0.13	0.13	0.13	0.13	
2							0.25	0.25	0.25	0.25	0.250	
4			0.56	0.53	0.52	0.51	0.50	0.50	0.50	0.50	0.50	

AmB MIC: 8 µg/mL
BA MIC: 1 mg/mL

Scedosporium aurantiacum **CBS 136046**

BA (columns) / AmB (rows)

µg/ mL	256	128	64	32	16	8	4	2	1	0.5	0.25	0.125
0.125												
0.25												
0.5												
1												
2												
4							0.07	0.06				
8							0.13	0.13				
16	0.51	0.38	0.31	0.28	0.27	0.26	0.25	0.25	0.25	0.25	0.25	
32	0.76	0.63	0.56	0.53	0.52	0.51	0.50	0.50	0.50	0.50	0.50	0.50

AmB MIC: 64 µg/mL
BA MIC: 1 mg/mL

Fusarium solani **SZMC 11524**

BA (columns) / AmB (rows)

µg/ mL	256	128	64	32	16	8	4	2	1	0.5	0.25	0.125
0.125												
0.25												
0.5												
1							0.50	0.50	0.50	0.50	0.50	0.50

AmB MIC: 2 µg/mL
BA MIC: 2 mg/mL

Fusarium solani **SZMC 11528**

BA (columns) / AmB (rows)

µg/ mL	256	128	64	32	16	8	4	2	1	0.5	0.25	0.125
0.125												
0.25												
0.5												
1								0.02	0.02	0.02	0.02	0.02
2						0.04	0.03	0.03	0.03	0.03	0.03	0.03
4					0.07	0.07	0.06	0.06	0.06	0.06	0.06	0.06
8			0.16	0.14	0.13	0.13	0.13	0.13	0.13	0.13	0.13	0.13
16		0.31	0.28	0.27	0.26	0.25	0.25	0.25	0.25	0.25	0.25	0.25
32	0.63	0.56	0.53	0.52	0.51	0.50	0.50	0.50	0.50	0.50	0.50	0.50

AmB MIC: 64 µg/mL
BA MIC: 2 mg/mL

FIG S1 Minimal Inhibitory Concentrations (MICs) and all of the tested combined application of Amphotericin B (AmB) and Betulinic Acid (BA) in case of 10 pathogenic fungal strains indicated with the FICI values and its effect in the cells. For the FICI values we interpreted FICI ≤ 0.5 as synergistic- (green), $0.5 < \text{FICI} \leq 1$ as additive- (blue), $1 < \text{FICI} \leq 4$ as indifferent- (orange) and FICI > 4 as antagonistic interactions. Grey color indicates those concentration combinations where the growth of the fungi was not inhibited.
